# Immune checkpoint status and oncogenic mutation profiling of rectal cancer after neoadjuvant chemotherapy (KSCC1301‐A2)

**DOI:** 10.1002/ags3.12730

**Published:** 2023-08-21

**Authors:** Yu Miyashita, Eiji Oki, Tomohiro Kamori, Yoshito Akagi, Shinichiro Mori, Norifumi Hattori, Kazuma Kobayashi, Mototsugu Shimokawa, Yoshinao Oda, Masaki Mori

**Affiliations:** ^1^ Department of Surgery and Science, Graduate School of Medical Science Kyushu University Fukuoka Japan; ^2^ Department of Anatomic Pathology, Graduate School of Medical Science Kyushu University Fukuoka Japan; ^3^ Department of Surgery Kurume University School of Medicine Kurume Japan; ^4^ Department of Digestive Surgery, Breast and Thyroid Surgery Kagoshima University Kagoshima Japan; ^5^ Department of Gastroenterological Surgery (Surgery II) Nagoya University Graduate School of Medicine Nagoya Japan; ^6^ Department of Surgery Nagasaki University Graduate School of Biomedical Sciences Nagasaki Japan; ^7^ Department of Biostatistics Yamaguchi University Graduate School of Medicine Yamaguchi Japan

**Keywords:** immune checkpoint inhibitor, neoadjuvant chemotherapy, next‐generation sequencing, rectal cancer, tumor microenvironment

## Abstract

**Aim:**

Immune checkpoint inhibitors (ICIs) are less effective in mismatch repair (MMR)‐proficient (pMMR) colorectal cancers (CRCs) than in MMR‐deficient CRCs. Here, we investigated changes in the tumor microenvironment after neoadjuvant chemotherapy (NAC) without radiotherapy in locally advanced rectal cancer (LARC) and the potential of ICIs as therapeutic agents for pMMR CRCs.

**Methods:**

This was an ad hoc analysis of a KSCC1301 randomized phase II trial in which patients with untreated resectable LARC were randomly assigned to receive S‐1 and oxaliplatin or folinic acid, 5‐fluorouracil, and oxaliplatin as NAC. Forty‐nine patients were studied in this ad hoc analysis. As a reference cohort, we assessed 25 rectal cancer patients who underwent surgery without NAC outside the randomized trial. Immune checkpoint molecules (ICMs; PD‐1, PD‐L1, CTLA‐4, LAG3), tumor‐infiltrating lymphocytes (TILs; CD8, FOXP3), and other related proteins were evaluated by immunohistochemistry. Next‐generation sequencing (NGS) using Oncomine™ Comprehensive Assay version 3 was conducted in 23 patients.

**Results:**

The expression levels of PD‐1, CTLA‐4, and LAG3 in the NAC group were significantly higher than in reference patients (*p* < 0.001). Additionally, the infiltration of CD8+ and FOXP3+ T cells, and the CD8/FOXP3 ratio were significantly higher in the NAC group than in reference patients (*p* < 0.0001). NGS analysis revealed no specific gene alteration related to TILs or ICMs.

**Conclusion:**

We demonstrated changes in the tumor immune microenvironment after NAC in pMMR rectal cancer. NAC was associated with increased expression of ICMs and TILs. Rectal cancer could be susceptible to combined immunotherapy with chemotherapy.

## Graphical abstract

We studied 49 patients with resectable locally advanced rectal cancer after neoadjuvant chemotherapy (NAC) without radiotherapy and 25 rectal cancer patients without NAC. Tumor immune microenvironment was significantly different between that observed after NAC without radiotherapy compared to the reference patients without NAC. NAC was associated with increased expression of immune checkpoint molecules and tumor‐infiltrating lymphocytes.

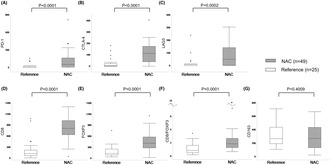

## 1 INTRODUCTION

Colorectal cancer (CRC) is one of the most commonly diagnosed cancers worldwide, and rectal cancer accounts for approximately 30% of all CRCs.^1^ Therapeutic strategies for colon and rectal cancers are different, and Western guidelines recommend preoperative (neoadjuvant) radiotherapy (RT) or chemoradiotherapy (CRT) for locally advanced rectal cancer (LARC).^2^, ^3^ Although these neoadjuvant therapies significantly reduce the incidence of local recurrence, no significant improvements of survival rates have been observed.^4^ Several clinical trials have investigated the effectiveness of neoadjuvant chemotherapy (NAC) alone for rectal cancer with oxaliplatin‐related regimens, but the long‐term oncological outcome data are unavailable for LARC.^5^, ^6^ The phase III Neoadjuvant folinic acid, 5‐fluorouracil, and oxaliplatin (mFOLFOX6) Chemotherapy With or Without Radiation in Rectal Cancer (FOWARC) trial compared neoadjuvant therapy with and without RT; the findings demonstrated that neoadjuvant mFOLFOX6 without RT resulted in lower rates of pathological complete response than that with RT.^7^ Long‐term follow‐up data, however, revealed no significant difference in disease‐free survival or local recurrence rate.^8^ NAC might be an option for treatment of LARC.

Immune checkpoint inhibitors (ICIs), targeting immune checkpoint molecules (ICMs) such as programmed death 1 (PD‐1), programmed death ligand 1 (PD‐L1), or cytotoxic T‐lymphocyte associated antigen 4 (CTLA‐4), are highly effective and have become the standard of care for patients with mismatch repair‐deficient (dMMR) or high microsatellite instability (MSI‐H) metastatic CRCs.^9^ In contrast, the same regimens have shown poor response rates in patients with mismatch repair‐proficient (pMMR) or microsatellite‐stable (MSS) metastatic CRCs.^10^ Although ICIs constitute an attractive therapy for microsatellite instability (MSI) patients, MSI is observed in only about 4%–13% of all CRCs.^11^, ^12^ MSI in rectal cancer is rarely noted. Hutchins et al. reported that the proportion of dMMR in rectal cancers was 1%.^13^

Recent studies have shown that tumor immune microenvironments are altered after NAC in several malignancies.^14^, ^15^ Similarly, upregulation of PD‐L1 or CD8+ T cells in rectal cancer patients after neoadjuvant CRT has been reported,^16^ suggesting the potential for a combined application of cytotoxic therapy and ICIs. In this study, we investigated changes in the tumor immune microenvironment after NAC without radiotherapy in patients with LARC and aimed to identify new biomarkers for ICIs.

## 2 MATERIALS AND METHODS

### 2.1 Patients and tissue samples

This study was conducted by the Kyushu Study Group of Clinical Cancer (KSCC) in Japan (KSCC1301‐A2). This was an ad hoc analysis of a KSCC1301 randomized phase II trial, in which Japanese patients with untreated resectable LARC were randomly assigned to receive S‐1 and oxaliplatin (SOX) or mFOLFOX6 as NAC.^17^ As a reference, we retrospectively assessed 37 Japanese rectal cancer patients outside the randomized trial who underwent surgery for primary rectal cancer without neoadjuvant therapy between January 2015 and December 2016 at the Department of Surgery and Sciences, Graduate School of Kyushu University Hospital. All tissue samples were obtained from resected specimens that included the invasive front of the tumor.

Pathological staging was performed according to the Union for International Cancer Control TNM classification (8th edition). All cases were reviewed based on imaging findings and histological examination. This trial was registered in the University Hospital Medical Information Network (UMIN) Clinical Trials Registry (UMIN0000031045). Written informed consent was obtained from the participants included in KSCC1301 randomized phase II trial.

### 2.2 Immunohistochemistry

Immunohistochemistry was performed in accordance with the manufacturer's recommendations. Primary antibodies and antigen retrieval for the immunohistochemical stains are described in Table S1. ICMs were evaluated by immunohistochemical staining for PD‐L1, PD‐1, CTLA‐4, and LAG3. We used CD8, FOXP3, and CD163 as markers for cytotoxic T lymphocytes, regulatory T lymphocytes, and M2‐type macrophages, respectively.

In CRC, true tumor cell PD‐L1 expression is rare, especially in pMMR cases. PD‐L1 expression is predominant on tumor infiltrating immune cells at the invasive front.^18^, ^19^ PD‐L1 was considered positive in tumor cells or tumor‐infiltrating mononuclear inflammatory cells in the stroma when membranous staining was evident in 1% or more of these cells.^20^ Cells positive for expression of PD‐1, CTLA‐4, LAG3, CD8, FOXP3, and CD163 were evaluated at 400× magnification in five independent fields at the invasive front.^21^ MMR status was assessed using immunohistochemical stains (MLH1, MSH2, MSH6, and PMS2). Expression of these stains was defined as absent when nuclear staining of tumor cells was completely absent in the presence of positive staining in surrounding cells. Assessment of HLA class I expression was performed according to the scoring system proposed by Ruiter et al.^22^ The intensity and percentage of cells in the tumor were determined based on the sum of the intensity of staining (ranging from 0 to 3) and the percentage of positive cells (ranging from 0 to 5). Loss of HLA class I was defined by a low score of 0–3. Microscopic interpretation of each immunohistochemical marker was performed independently by two investigators (T.K. and Y.M.).

### 2.3 Next‐generation sequencing analysis

All genomic analyses were performed by next‐generation sequencing (NGS) using Oncomine™ Comprehensive Assay (OCA) version 3 (OCAv3; Thermo Fisher Scientific). OCAv3 is a targeted NGS panel that detects 161 genes relevant to solid tumors (Table S2). We commissioned Takara Bio Inc. (Shiga) to perform DNA and RNA extraction, library preparation, sequence analysis, and sequence data analysis. Among the candidate gene variants, we incorporated an analysis filter selecting only those variants with >50 reads and allelic frequency >3%. Mutations with known clinical significance were identified by comparing variants against the ClinVar database. Only functional consequences of variants that were classified as pathogenic or likely pathogenic were selected for analysis. For copy number alterations, we incorporated an analysis filter selecting only copy number amplifications greater than 2.5. Mutation and copy number data were analyzed and mapped using the Bioconductor and maftools package in R software.

### 2.4 Statistical analysis

Patient characteristics were compared between two groups using Fisher's exact test or chi‐squared test. The relationships of continuous variables between or among groups were compared using Wilcoxon's rank‐sum test. A *p* < 0.05 was considered statistically significant. All statistical analyses were performed with JMP pro 14 software (SAS Institute Inc.).

## 3 RESULTS

### 3.1 Patient characteristics

**FIGURE 1:**
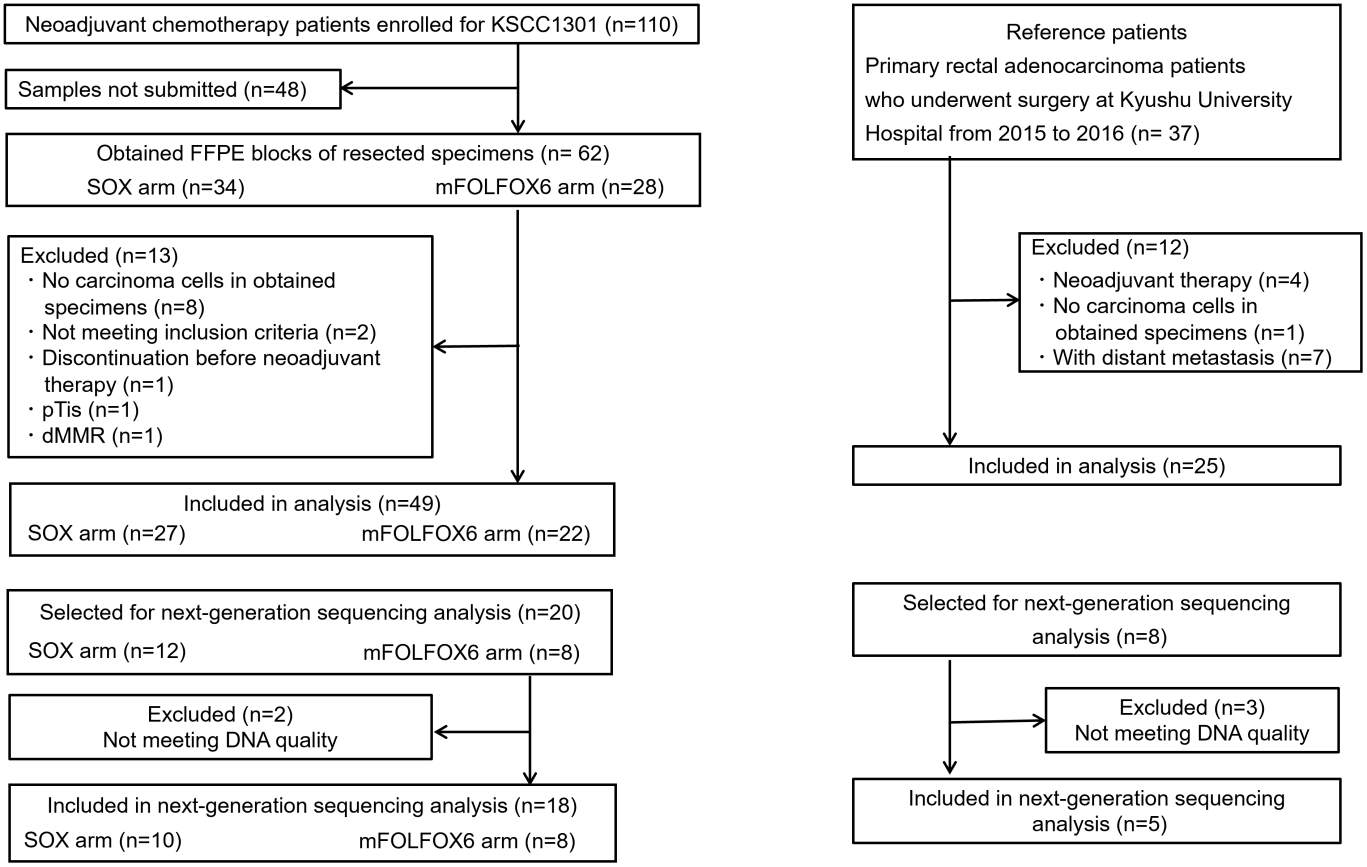
The flowchart for the cohort study. Full analysis set was eventually based on 49 patients for the neoadjuvant therapy (NAC) group and 25 patients for the reference group.

The study flowchart is shown in Figure 1. Forty‐nine patients who were enrolled for the KSCC1301 study were included in this ad hoc analysis. The patients underwent surgery from April 2014 to February 2016. As a reference, 25 patients with rectal cancer who underwent surgery without neoadjuvant therapy were assessed outside the randomized trial. Clinicopathological characteristics of ad hoc analysis are shown in Table 1. There were no significant differences clinically or pathologically between the SOX and mFOLFOX6 arms. Clinicopathological characteristics of patients receiving NAC and the reference patients are presented in Table 2. The number of clinical lymph node metastases in patients receiving NAC was higher than in the reference patients. The number of pathological lymph node metastases, however, was higher in the reference patients than in patients receiving NAC.

**TABLE 1:** Clinicopathological characteristics of patients receiving neoadjuvant chemotherapy.

Factor	SOX		mFOLFOX6		*p* value
	*n* = 27	%	*n* = 22	%	
Age					
Median	63		64		0.7171
Range	37–79		36–78		
Sex					
Male	21	77.8	19	86.4	0.4876
Female	6	22.2	3	13.6	
Depth of wall invasion					
~T3	25	92.6	20	90.9	1.0000
T4	2	7.41	2	9.1	
Lymph nodes metastasis					
Yes	4	14.8	8	36.4	0.1037
No	23	85.2	14	63.6	
Distant metastasis					
Yes	0	0.00	0	0.00	—
No	27	100.0	22	100.0	
Lymph vessel invasion					
Yes	10	37.0	8	36.4	1.0000
No	17	63.0	14	63.6	
Venous invasion					
Yes	12	44.4	11	50.0	0.7777
No	15	55.6	11	50.0	
Histological type					
Well/moderately	26	96.3	21	95.5	1.0000
Poorly/others	1	3.7	1	4.5	
Therapeutic effect					
Grade 0–1	23	85.2	16	72.7	0.2669
Grade 2	3	11.1	6	27.3	
Unknown	1	3.7	0	0.0	

**TABLE 2:** Clinicopathological characteristics of patients in the neoadjuvant chemotherapy (NAC) and reference groups.

Factor	NAC		Reference		*p* value
	*n* = 25	%	*n* = 25	%	
Age					
Median	63		65		0.4133
Range	36–79		33–83		
Sex					
Male	40	81.6	17	68	0.2446
Female	9	18.4	8	32	
Depth of wall invasion (clinical)					
~T3	33	67.4	22	88	0.0897
T4	16	32.7	3	12	
Depth of wall invasion (pathological)					
~T3	45	91.8	25	100	0.2931
T4	4	8.2	0	0	
Lymph nodes metastasis (clinical)					
Yes	32	65.3	7	28	0.0032
No	17	34.7	18	72	
Lymph nodes metastasis (pathological)					
Yes	12	24.5	13	52	0.0221
No	37	75.5	12	48	
Distant metastasis					
Yes	0	0	0	0	—
No	49	100	25	100	
Lymph vessel invasion					
Yes	18	36.7	9	36	1
No	31	63.3	16	64	
Venous invasion					
Yes	23	46.9	9	36	0.4595
No	26	53.1	16	64	
Histological type					
Well/moderately	47	95.9	25	100	0.5465
Poorly/others	2	4.1	0	0	

### 3.2 Immunohistochemical findings

**FIGURE 2:**
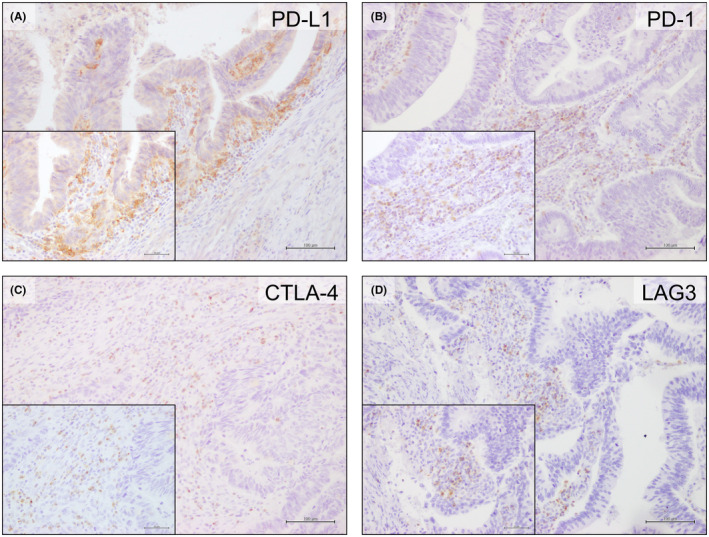
Representative images of the expression of PD‐L1 (A), PD‐1 (B), CTLA‐4 (C), and LAG3 (D). Membrane staining was conducted for PD‐L1 and PD1. Membrane and cytoplasmic staining were carried out for CTLA‐4 and LAG3. Scale bars: 100 and 50 μm. PD‐L1, programmed death ligand 1; PD‐1, programmed death 1; CTLA‐4, cytotoxic T‐lymphocyte associated antigen 4; LAG3, lymphocyte activation gene 3.

**FIGURE 3:**
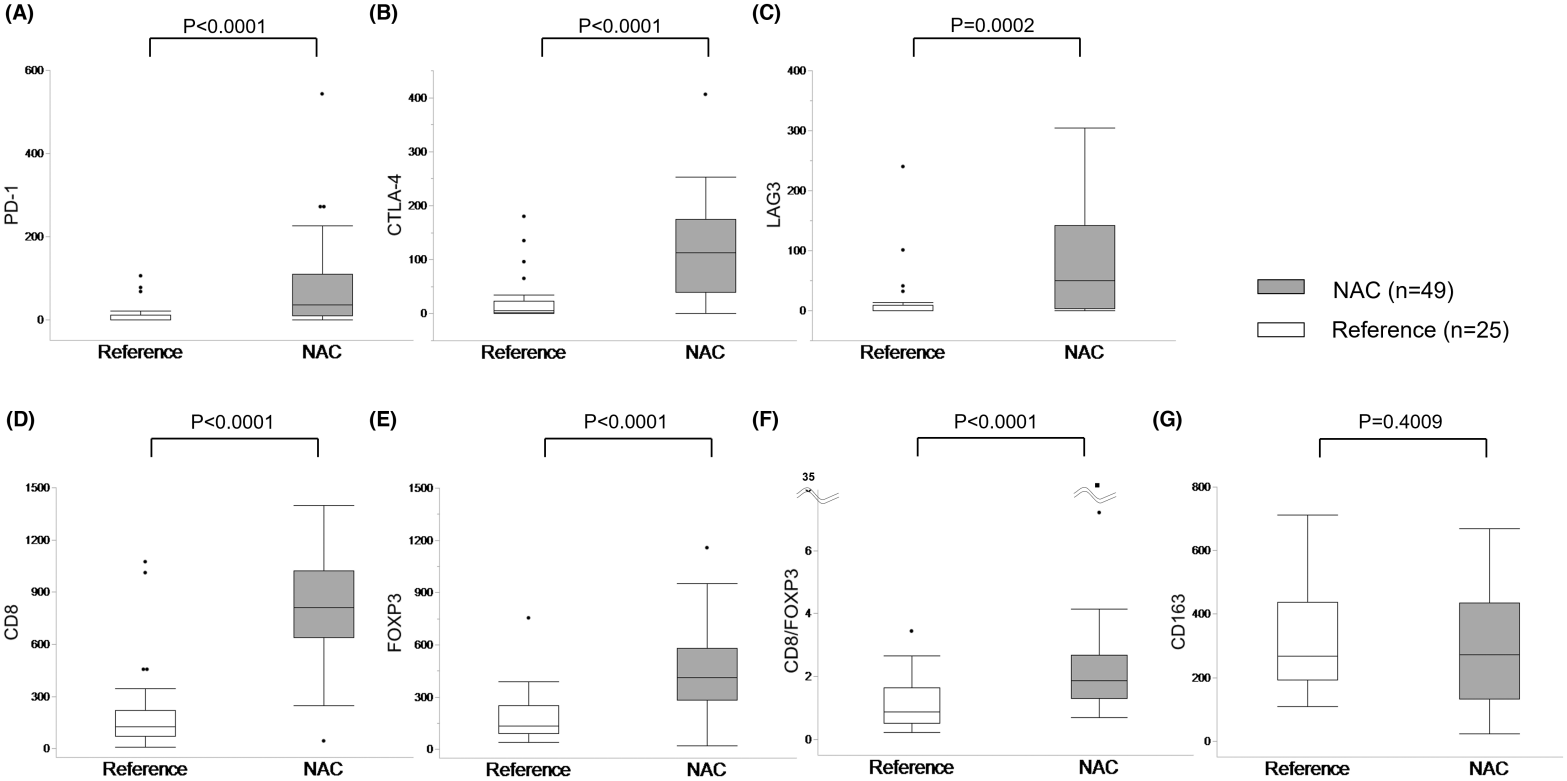
Comparison of expression for PD‐1 (A), CTLA‐4 (B), LAG3 (C), CD8 (D), FOXP3 (E), CD8/FOXP3 (F), and CD163 (G) between the NAC (*n* = 49) and reference (*n* = 25) groups.

Representative images of immunohistochemical staining of ICMs (PD‐L1, PD‐1, CTLA‐4, and LAG3) are shown in Figure 2. Expression levels of ICMs were not significantly different between the SOX and mFOLFOX6 arms (Figure S1). Expression levels of PD‐1, CTLA‐4, and LAG3 in the NAC group were significantly higher than those in the reference cohort (*p* < 0.0001, *p* < 0.0001, and *p* = 0.0002, respectively; Figure 3A–C). When the cut‐offs for defining PD‐1, CTLA‐4, and LAG3 expression were set at the median, there were also significant differences between the NAC and reference groups (Table 3). There were no significant differences between the SOX and mFOLFOX6 arms (Table S3). Expression levels of PD‐L1 were relatively higher in patients receiving NAC than in the reference patients, but not significantly different (*p* = 0.2171; Table 3). There were only two cases that showed loss of HLA class I expression (Table 3, Table S3).

**TABLE 3:** Immunohistochemical results of ICMs, MMR‐related proteins, and HLA class I.

Factor	Neoadjuvant chemotherapy		Reference		*p* value
	*n* = 49	%	*n* = 25	%	
PD‐L1					
Positive	30	61.2	14	56	0.2171
Negative	19	38.8	11	44	
PD‐1					
Positive	34	69.4	4	16	<0.0001
Negative	15	30.6	21	84	
CTLA‐4					
Positive	33	67.4	4	16	<0.0001
Negative	16	32.7	21	84	
LAG3					
Positive	32	65.3	5	20	0.0004
Negative	17	34.7	20	80	
MMR					
Deficient	0	0	0	0	—
Proficient	49	100	25	100	
HLA class I					
Loss	1	2	1	4	1
Retain	48	98	24	96	

Abbreviations: HLA, human leukocyte antigen; ICMs, immune checkpoint molecules; MMR, mismatch repair.

We then analyzed tumor‐infiltrating lymphocyte (TILs) and macrophages. We used CD8, FOXP3, and CD163 as markers for cytotoxic T lymphocytes, regulatory T lymphocytes, and M2‐type macrophages, respectively. There were no significant differences between the SOX and mFOLFOX6 arms (Figure S2). Infiltration of CD8+ and FOXP3+ T cells was significantly higher in patients receiving NAC than in the reference patients (*p* < 0.0001; Figure 3D,E). We evaluated the CD8/FOXP3 ratio; it was also significantly higher in patients receiving NAC than in the reference patients (*p* < 0.0001; Figure 3F), but there was no significant difference between the SOX and mFOLFOX6 arms (Figure S2C). CD163+ cells demonstrated no significant difference between the NAC and reference groups (*p* = 0.2827; Figure 3G).

### 3.3 Next‐generation sequencing

NGS was used to analyze 20 cases from the NAC group and eight cases from the reference group. Five cases were excluded from analysis due to the low quality or quantity of DNA. Consequently, 18 cases from the NAC group and five cases from the reference group were included in the analysis (Figure 1). Clinicopathological characteristics and immunohistochemical results for CD8 and PD‐1 of the patients included in NGS analysis are presented in Table S4. The cut‐offs for defining CD8 and PD‐1 expression were set at the median for each group (NAC or reference).

**FIGURE 4:**
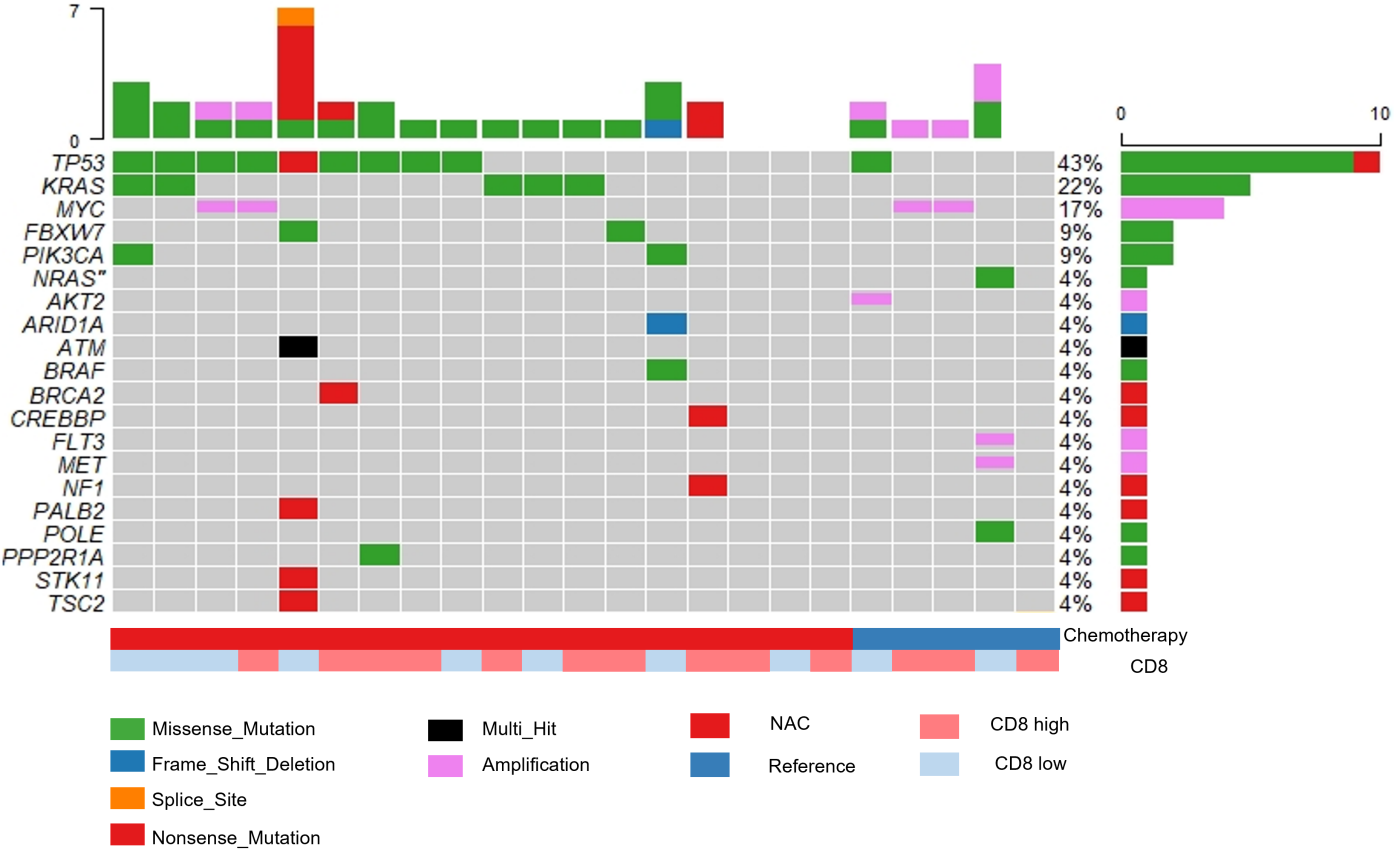
Summary of the molecular characteristics of next‐generation sequencing analysis (*n* = 23). The columns in the table denote samples, and the rows denote genes. The right and top sides of the graphic represent the number of patients in whom each gene alteration was detected.

Oncoplots for detected gene mutations or copy number amplifications for the sequence are shown in Figure 4. Among 23 cases, we identified 32 mutations in 16 genes and seven copy number amplifications in four genes, with at least one alteration being present in 82.6% (19 out of 23 cases). The frequently called variants included TP53 mutations (10 cases, 43.5%), followed by KRAS mutations (five cases, 21.7%), MYC amplifications (four cases, 17.3%), and PIK3CA mutations (two cases, 8.7%). In the NAC group, frequently called variants included TP53 mutations (nine out of 18 cases, 50.0%), followed by KRAS mutations (five cases, 27.8%), PIK3CA mutations (two cases, 11.1%), and MYC amplifications (two cases, 11.1%).

We then evaluated the number of detected gene mutations that showed no significant difference between the NAC and reference groups (*p* = 0.1098; Figure S3A). Regarding the infiltration of CD8+ lymphocytes, the number of mutant genes was relatively lower in the CD8‐high group than the CD8‐low group (*p* = 0.0505; Figure S3B). A higher sequencing failure rate was observed with the RNA panel than DNA.

In addition, we compared the tumor immune microenvironment according to gene status and chemotherapy status. Mutations in genes in the growth factor signal cascade (RAS/RAF/MAP kinase; i.e., AKT1, AKT2, ALK, BRAF, EGFR, ERBB2, ERBB3, FGFR, KRAS, MET, NRAS, NTRK1, PTEN, PIK3CA, RET) were detected in six (26.1%) cases in the NAC group and two (8.7%) cases in the reference group. There were no associations between RAS/RAF/MAP kinase status and the tumor immune microenvironment in the NAC group (*n* = 18; Figure S4).

Among RAS/RAF/MAP kinase mutant cases (*n* = 8), PD‐1 expression and infiltration of CD8+ and FOXP3+ T cells were significantly higher in the NAC group than in the reference group (Figure S5). Among wild‐type RAS/RAF/MAP kinase cases (*n* = 15), there were no significant differences between the NAC and reference groups (Figure S6). Collectively, NGS analysis revealed no specific gene alteration related to TILs or ICMs. However, mutations in the RAS/RAF/MAP kinase pathway had some impact on the infiltration of CD8+ and FOXP3+ T cells.

## 4 DISCUSSION

In the present study, we demonstrated that the expression of ICMs and TILs was significantly changed after NAC in patients with rectal cancer. Shinto et al. proposed that CD8+ T cells were enhanced after neoadjuvant CRT in patients with rectal cancer, an increase triggered by the CRT‐mediated release of tumor‐associated antigens.^23^ Some studies implied that direct irradiation of tumor tissue upregulates and releases tumor‐associated antigens and induces immunogenic cell death that in turn activates cytotoxic T lymphocytes.^24^ Immunogenic cell death can be induced by chemotherapy using anthracycline or oxaliplatin.^24^, ^25^ In this study, two oxaliplatin‐based regimens, SOX and mFOLFOX6, were used for NAC. Although the expression of CD8+ T cells was not significantly different between the SOX and mFOLFOX6 arms, the infiltration of CD8+ T cells in patients receiving NAC was significantly higher than that in the reference cohort. This may indicate immunogenic cell death was induced by NAC, resulting in upregulated CD8+ T cell infiltration.

Regarding immune suppressive cells, our results showed that the numbers of FOXP3+, PD‐1+, CTLA‐4+, and LAG3+ cells in the NAC group were significantly higher than in the reference group. PD‐L1 expression was relatively higher in the NAC group. One possible mechanism underlying this response is feedback caused by increased numbers of CD8+ T cells. FOXP3+ regulatory T cells have been reported to be driven by CD8+ T cells via the production of chemokine receptor type 4 (CCR4)‐binding chemokines.^26^ Some studies have reported that upregulation of PD‐L1 depended on CD8+ T cell infiltration, and expression of PD‐L1 was induced by interferon‐γ derived from TILs.^14^, ^27^ CTLA‐4 and LAG3 are immune checkpoint molecules that are upregulated following T cell activation.^28^ Likewise, PD‐1 is expressed on activated (but not resting) T cells. PD‐1 is induced late after activation following expression of CTLA‐4.^29^ The present study suggests that the T cell immune response may be activated by NAC.

Another possible mechanism is that PD‐1/PD‐L1 is upregulated to help cancer cells escape from immune‐mediated cell death. PD‐L1 is expressed on immunogenic tumor cells and permits them to escape from the host T cell immunity.^30^ It is possible that enhanced PD‐1/PD‐L1 in patients receiving NAC may be a result of escape from immune‐mediated cell death.

Recent studies have proposed that cancer cells can be eliminated by host cytotoxic CD8+ T cells and that tumor infiltrating CD8+ T cells are a predictive biomarker for ICIs.^31^ In addition, a high CD8/FOXP3 ratio may correlate with improved prognosis.^32^ Our results showed that tumor‐infiltrating CD8+ T cells, the CD8/FOXP3 ratio, and ICMs were significantly higher in patients receiving NAC. That finding suggests that the tumor immune microenvironment was made more immunogenic by NAC. This result suggests that combining immunotherapy with chemotherapy may be effective in patients with rectal cancer. Sequential therapy, i.e., NAC as induction therapy followed by ICI, may be effective. Additionally, concurrent therapy with chemotherapy plus ICI may have similar effects.

We also conducted an NGS analysis in this study. The most frequently detected gene mutations were in TP53, followed by KRAS and PIK3CA mutations. This result was similar to previous reports for patients with left‐sided CRC.^33^ In the present study, the number of detected gene mutations was relatively lower in CD8‐high than CD8‐low patients. Meanwhile, the number of detected gene mutations was not significantly different between the NAC and reference groups. Recent studies have proposed that driver mutations, such as EGFR mutations or RHOA mutations, are associated with the immunosuppressive tumor microenvironment.^34^, ^35^ In the present study, we incorporated into the analysis a filter for depth or allelic frequency from targeted genes relevant to solid tumors. Furthermore, we selected only functional consequences of variants that are classified as pathogenic. The tumors with driver mutations may possibly be correlated with suppressive tumor immune microenvironments.

Collectively, our results suggest that in the absence of specific mutations, the tumor immune microenvironment was made more immunogenic by NAC, suggesting that combining immunotherapy with chemotherapy may be effective in patients with pMMR rectal cancer.

This study had some limitations. First, the tumor immune microenvironments of cases were not compared before and after NAC. Pretreatment biopsy specimens were not evaluated in this study. The expression of ICMs and TILs was evaluated by resected specimens at the invasive front, and we did not compare these with the biopsy specimens before NAC. Second, clinicopathological characteristics between patients receiving NAC and the reference patients were statistically different. In clinical analyses, patients receiving NAC showed more lymph node metastases than the reference patients, although the opposite result was observed in pathological analyses. Clinical T stage analysis also revealed higher cT4 in patients receiving NAC than in the reference patients, although this difference was not significant. This result may reflect that patients receiving NAC had more aggressive diseases in the clinical analyses. Pathological difference in lymph node metastases may be due to treatment effects. Third, NGS analysis was not conducted for all cases. Although we compared the number of detected gene mutations, we could not compare tumor‐mutation burden due to the NGS panel. The number of cases with gene mutations is small, which may be insufficient for statistical analysis. Additionally, a validation cohort was not conducted to further confirm the immunohistochemistry or NGS findings.

In conclusion, we demonstrated a change in the tumor immune microenvironment after NAC in pMMR rectal cancer. NAC was associated with increased expression of ICMs and TILs. We suggest that rectal cancer could be treated with a combination of immunotherapy and chemotherapy.

## AUTHOR CONTRIBUTIONS

YM and EO drafted the manuscript. EO and MM were involved in conception and design. YM, TK, and EO were involved in analysis and interpretation of data. YA, SM, NH, and KK were involved in data collection. YM, TK, and MS were involved in statistical analysis. EO, YO, and MM supervised the study. All authors contributed to the discussion and revision of the manuscript.

## FUNDING INFORMATION

This study was funded by Ono Pharmaceutical Co. Ltd. under a research contract and supported by KSCC (Kyushu Study Group of Clinical Cancer), which provided assistance with the collection, analysis, and interpretation of data.

## CONFLICT OF INTEREST STATEMENT

Eiji Oki has received honoraria for lecturing from Eli lily, Bayer, Ono Pharmaceutical Co., Ltd., Chugai Pharmaceutical Co., Ltd., Taiho Pharmaceutical Co., Ltd., Takeda Pharmaceutical Co., Ltd., Bristol Myers squibb. Other authors have no relevant financial or non‐financial interests to declare. Eiji Oki and Masaki Mori are the editorial board members of *Annals of Gastroenterological Surgery*. The authors declare no conflict of interests for this article.

## ETHICS STATEMENT

This study was performed in line with the principles of the Declaration of Helsinki. Approval was granted by the Institutional Review Board of Kyushu University, Fukuoka, Japan (2019‐201, 29‐298, 29‐509).

## Supporting information


Figure S1.

Table S1.

## Data Availability

The datasets generated during and/or analyzed during the current study are available from the corresponding author on reasonable request.
