# Supplementary material for: Immune checkpoint status and oncogenic mutation profiling of rectal cancer after neoadjuvant chemotherapy (KSCC1301‐A2)
Source: Ann Gastroenterol Surg. 2023 Aug 21;8(2):251–61. doi: 10.1002/ags3.12730 (PMC10914707; doi:10.1002/ags3.12730)
Supplement: Supplementary file 1 — Figure S1. [file AGS3-8-251-s001.pptx]

## Slide 1
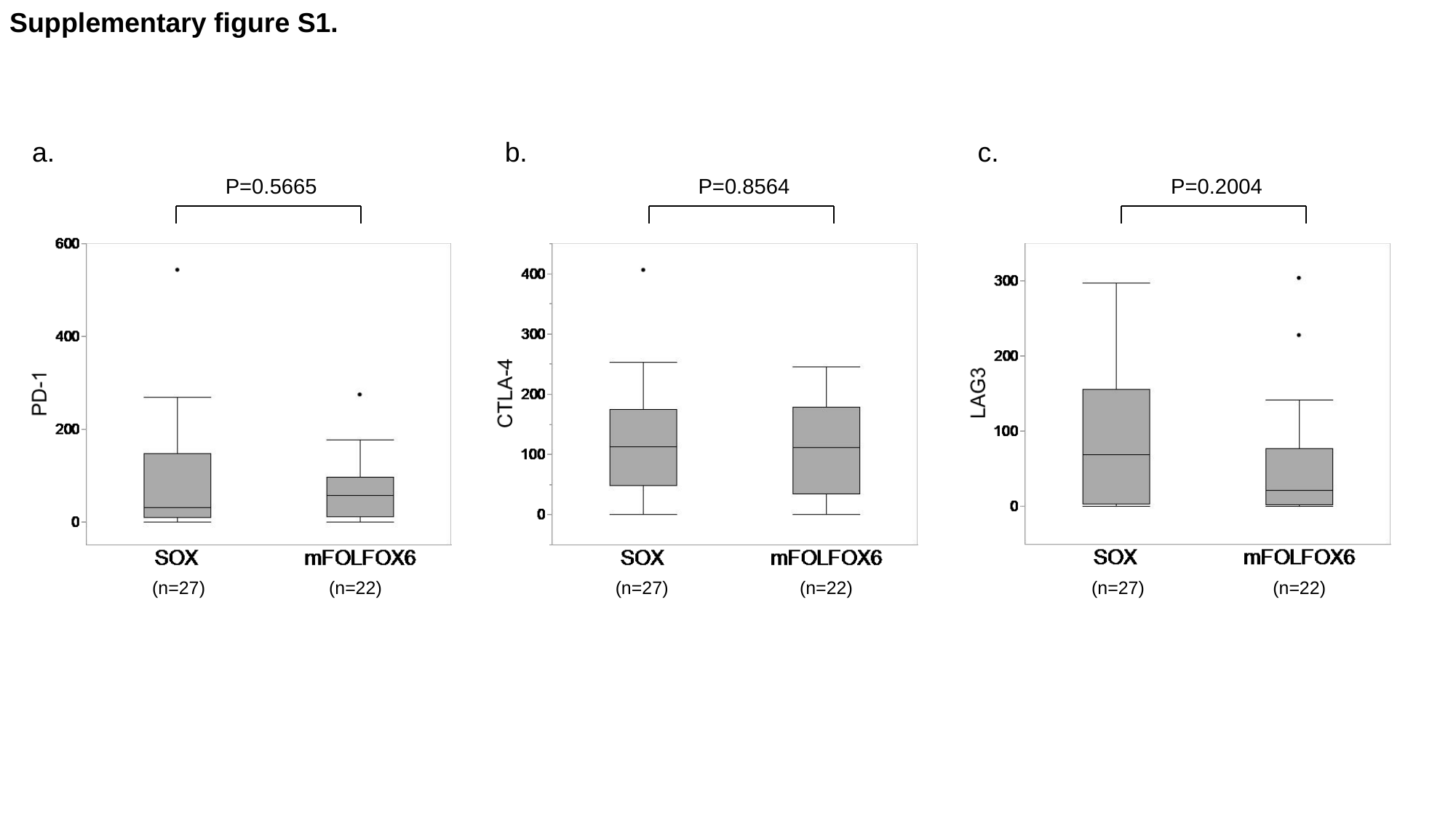

Supplementary figure S1.
a.
b.
c.
P=0.5665
P=0.8564
P=0.2004
(n=27)
(n=22)
(n=27)
(n=27)
(n=22)
(n=22)

## Slide 2
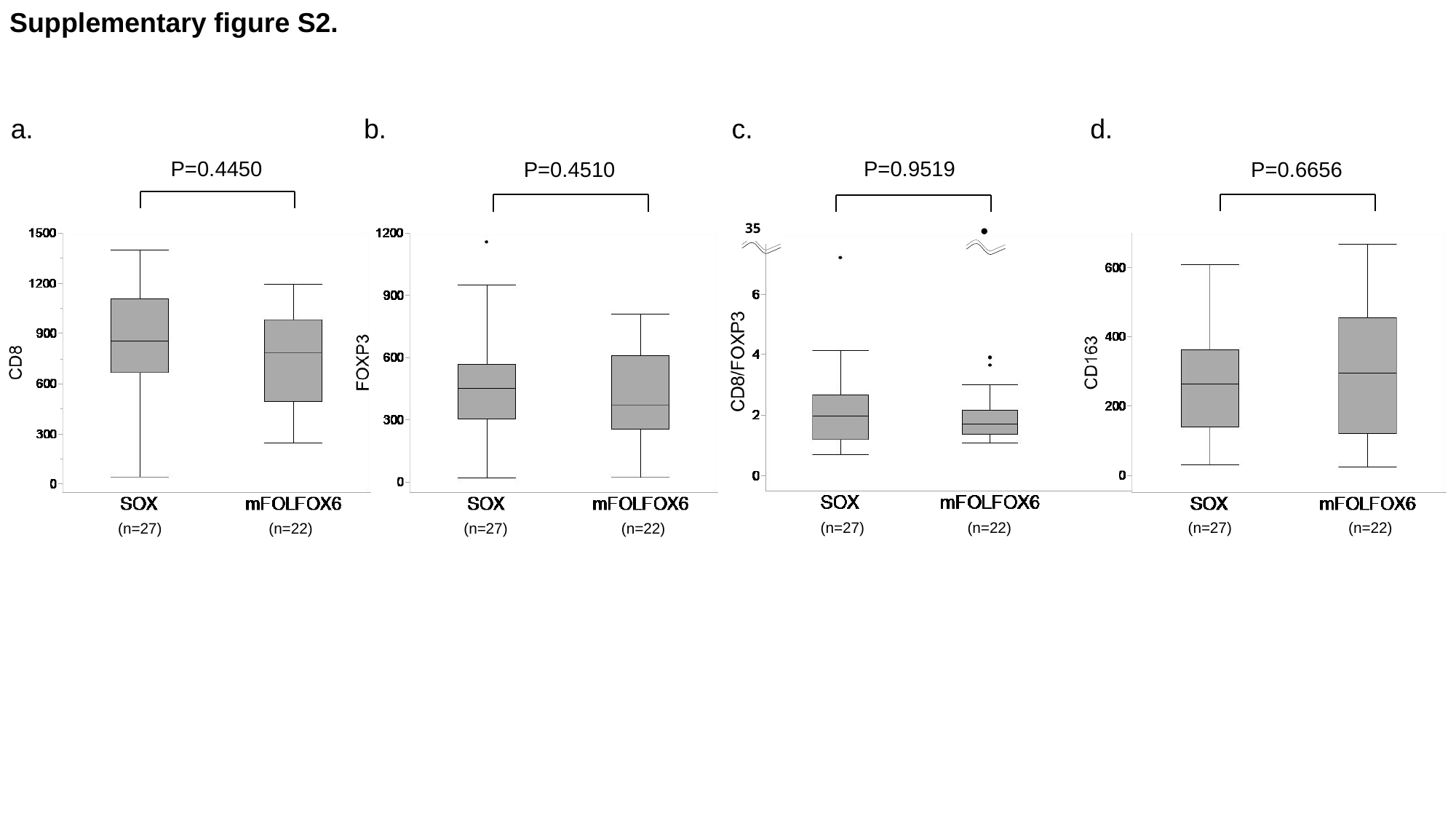

Supplementary figure S2.
a.
b.
c.
d.
P=0.4450
P=0.9519
P=0.6656
P=0.4510
・
35
(n=27)
(n=22)
(n=27)
(n=22)
(n=22)
(n=27)
(n=27)
(n=22)

## Slide 3
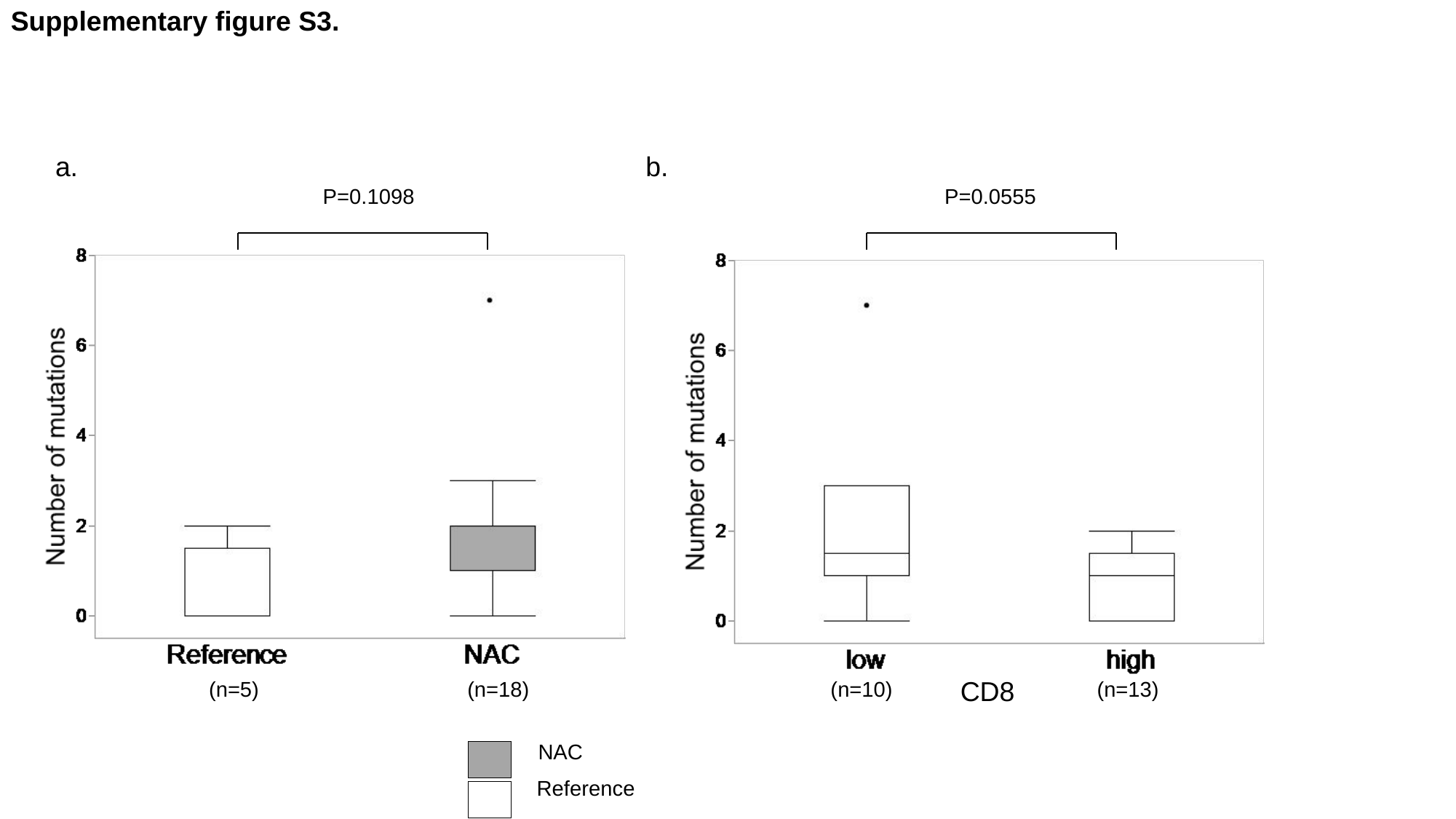

Supplementary figure S3.
a.
b.
P=0.0555
P=0.1098
CD8
(n=5)
(n=18)
(n=10)
(n=13)
NAC
Reference

## Slide 4
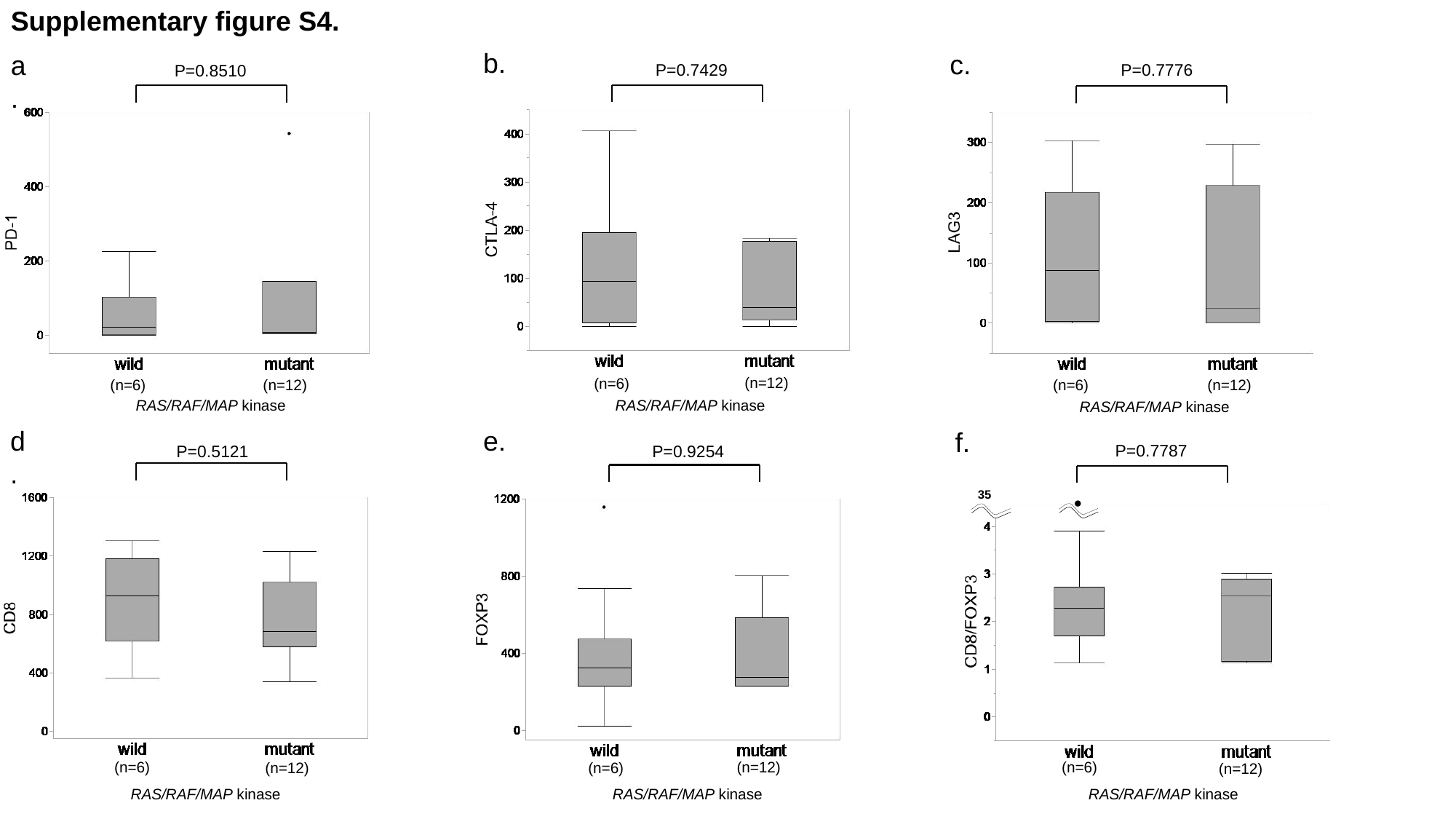

Supplementary figure S4.
b.
c.
a.
P=0.7429
P=0.7776
P=0.8510
(n=12)
(n=6)
(n=6)
(n=12)
(n=6)
(n=12)
RAS/RAF/MAP kinase
RAS/RAF/MAP kinase
RAS/RAF/MAP kinase
e.
d.
f.
P=0.7787
P=0.5121
P=0.9254
・
35
(n=6)
(n=12)
(n=6)
(n=6)
(n=12)
(n=12)
RAS/RAF/MAP kinase
RAS/RAF/MAP kinase
RAS/RAF/MAP kinase

## Slide 5
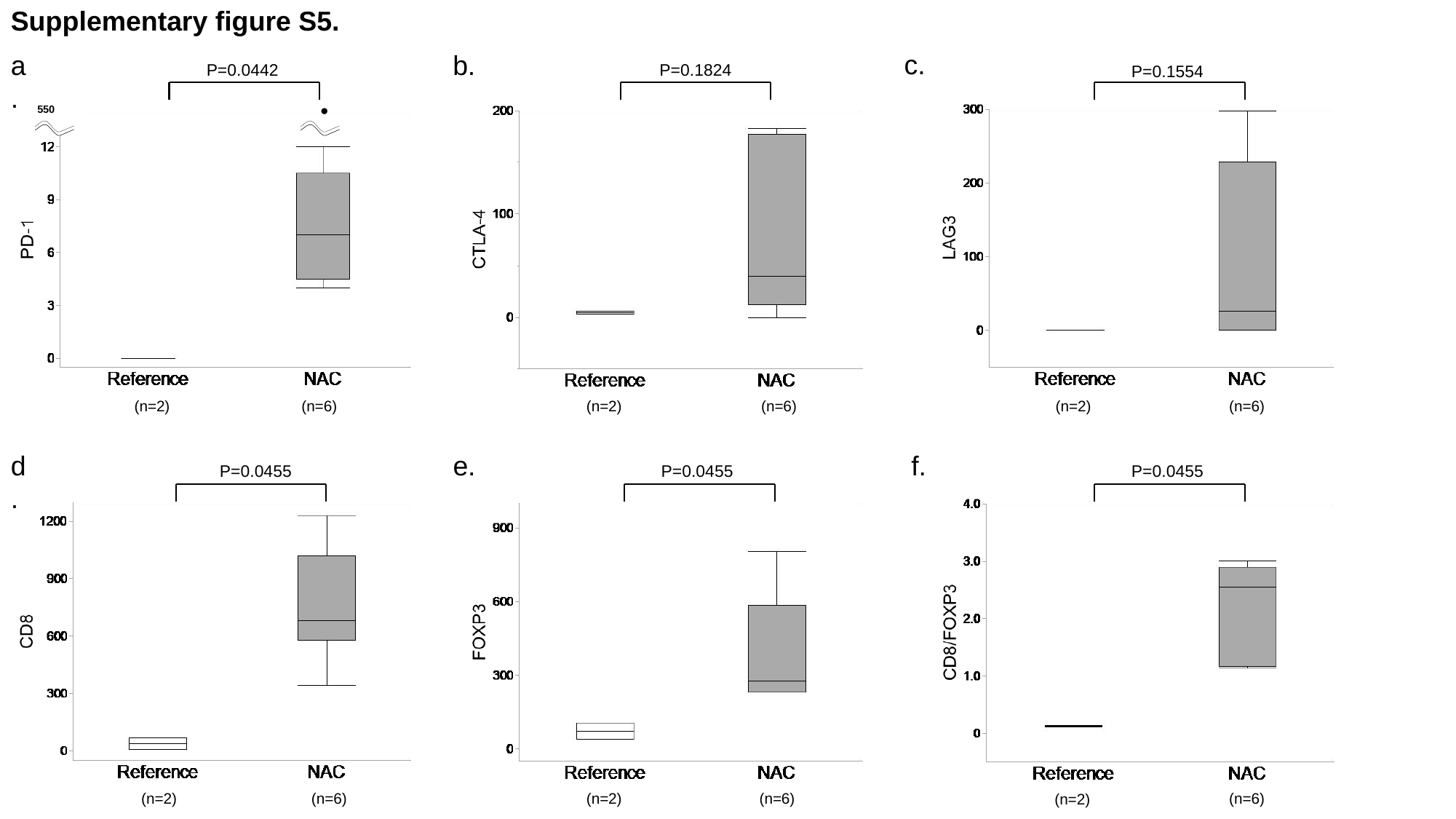

Supplementary figure S5.
c.
a.
b.
P=0.0442
P=0.1824
P=0.1554
・
550
(n=2)
(n=6)
(n=2)
(n=6)
(n=2)
(n=6)
d.
e.
f.
P=0.0455
P=0.0455
P=0.0455
(n=6)
(n=2)
(n=6)
(n=2)
(n=6)
(n=2)

## Slide 6
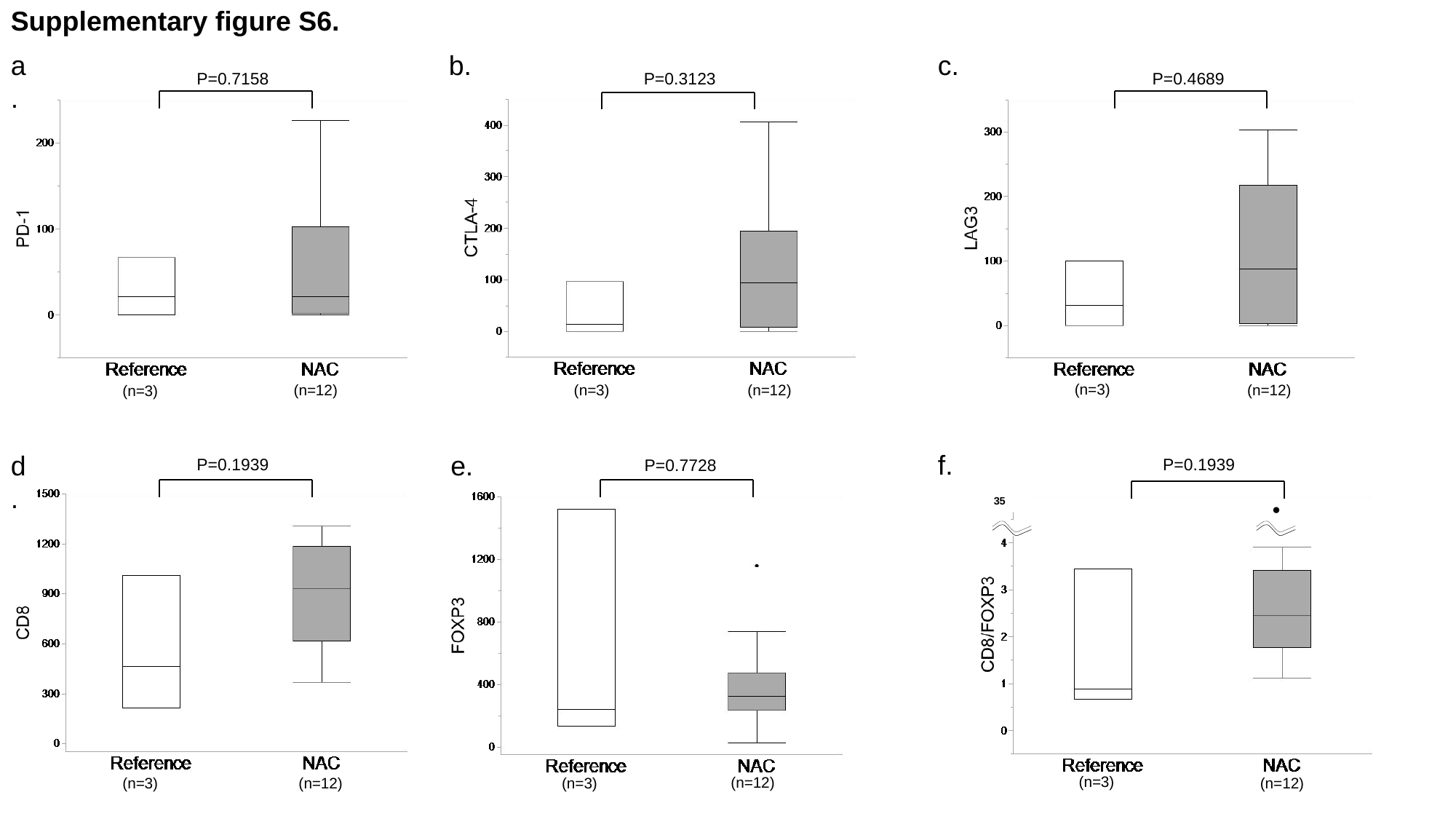

Supplementary figure S6.
a.
b.
c.
P=0.7158
P=0.4689
P=0.3123
(n=3)
(n=12)
(n=3)
(n=12)
(n=12)
(n=3)
f.
d.
e.
P=0.1939
P=0.1939
P=0.7728
・
35
(n=3)
(n=12)
(n=3)
(n=12)
(n=3)
(n=12)
